# Biological Mechanisms behind Wischnewsky Spots Finding on Gastric Mucosa: Autopsy Cases and Literature Review

**DOI:** 10.3390/ijerph19063601

**Published:** 2022-03-18

**Authors:** Matteo Antonio Sacco, Ludovico Abenavoli, Cristina Juan, Pietrantonio Ricci, Isabella Aquila

**Affiliations:** 1 Institute of Legal Medicine, Department of Medical and Surgical Sciences, University “Magna Graecia” of Catanzaro, 88100 Catanzaro, Italy; matteoantoniosacco@gmail.com (M.A.S.); ricci@unicz.it (P.R.); 2 Department of Health Sciences, University “Magna Graecia” of Catanzaro, 88100 Catanzaro, Italy; l.abenavoli@unicz.it; 3 Laboratory of Food Chemistry and Toxicology, Faculty of Pharmacy, University of Valencia, Burjassot, 46100 València, Spain; cristina.juan@uv.es

**Keywords:** Wischnewsky spots, stomach, autopsy, hypothermia, biology, agony, ketoacidosis

## Abstract

Hypothermia is an emergency caused by the lowering of the central body temperature with a slowdown of basic vital functions. Reduced mobility, old age, psychiatric or metabolic disorders are relevant risk factors. Diagnosis of death from hypothermia is a challenge, as there are no pathognomonic signs, and supportive findings can be inconstant. Wischnewsky Spots (WS) are blackish lesions of gastric mucosa, typically associated with hypothermic death. The pathophysiology of these lesions is still uncertain. The aim of this paper is to investigate the pathological mechanisms determining the appearance of WS by analyzing the current scientific knowledge in this area. We performed a narrative review of the literature published in the last 20 years, comparing the results with three cases of hypothermia reported from our experience. The review proved that WS show a multifactorial etiology, i.e., not only body temperature decrease, but also various extrinsic and intrinsic factors, such as physical and psychological stress, agony, *causa mortis* and metabolic comorbidities. The review summarizes the current knowledge in the field of incidence, pathology and morphology of WS by proposing some scientific and technical points for clinical and forensic analysis of this phenomenon.

## 1. Introduction

Hypothermia is a reduction in central body temperature < 35 °C caused by the alteration of thermoregulation mechanisms, which is generally due to immersion in cold water or staying in cold environments for long time [1]. Risk factors include trauma resulting in the reduced mobility, low body mass index, intoxication from alcohol, drugs, psychotropic drugs, psychiatric or metabolic disorders. Clinical symptoms of hypothermia include lethargy, hallucination and neurological complications, such as loss of consciousness up to coma with death. The pathophysiology of hypothermic death depends on the progressive slowdown of the primary physiological functions of the nervous, cardiovascular and respiratory systems with fatal arrhythmias up to cardiac arrest. In addition, the body temperature decrease is associated with a reduction in the levels of antidiuretic hormone (ADH) with increased diuresis, loss of fluids from interstitial tissues and hypovolemia with vasoconstriction [2].

Post-mortem diagnosis of hypothermia is a challenge, due to the lack of pathognomonic signs. Several authors have reported some external body signs, such as pink discoloration of the skin on joints (frost erythema), and internal body signs, including Wischnewsky spots (WS), hemorrhage of the synovial membranes, hemorrhages of the skeletal muscles (especially iliopsoas), vacuolization of the renal tubular epithelial cells, pancreas inflammation or necrosis (due to rewarming) [3]. However, these signs may be inconsistent or completely absent, so that, in many cases, a forensic pathologist may encounter serious difficulties in diagnosis with relevant medico-legal implications. Diagnosis may be more difficult when concomitant traumas are found, or post-mortem injuries caused by prolonged immersion in water mask the real cause of death. Furthermore, in several cases, the pathologist may not have data about the body temperature at the time of death, and the local environmental temperature will also often not be known. Additionally, post-mortem cooling/refrigeration might have relevant effects on the appearance of the body at autopsy. For instance, frost erythema can arise due to cooling in the mortuary refrigerator.

Among the most relevant signs, the so-called Wischnewsky spots (WS) were reported on the stomach mucosa. The name derives from Dr SM Wischnewsky who, toward the end of 1800, reported these spots in over 90% of hypothermia cases, describing them as hemorrhages of the gastric mucosa, of variable number and size, with oval or point-like shape [3]. Subsequently, several authors confirmed WS finding in cases of fatal hypothermia, proposing different hypotheses with controversial mechanisms. WS still remain a fascinating phenomenon with uncertain incidence, pathophysiology and diagnostic value. The purpose of this review is to propose a synthetic but organic view of literature about WS, investigating the biological factors influencing their appearance. Gross and microscopic characteristics with proposed hypotheses in the literature are discussed. The review results are compared with three forensic autopsies with WS finding reported from our experience.

## 2. Materials and Methods

### 2.1. Forensic Investigations

In all three reported cases, investigators performed a scene analysis of where the body was found, and photographs were taken. On the scene, entry and exit points of the scene were analyzed, and the possible routes taken were evaluated. Camera images were acquired, if available. The ambient and body temperature were measured, and meteorological conditions in the previous days were evaluated. Information on age and comorbidities was collected through the analysis of medical records. The type of psychiatric illness, drug treatment and any previous escape attempts were assessed. An autopsy was carried out in all cases. Topography and size of every injury was evaluated at external examination. All organs were photographed, examined, and representative samples of each organ were fixed in 10% formalin for histological microscopic investigation. Particular attention was paid to the analysis of the gastric mucosa through the macroscopic examination of the spots, with measurement of the lesions and sampling of the areas of interest. Then, biological fluids were taken, including blood, vitreous humor and urine, for toxicological investigations. Fluids were analyzed with an automated immunoassay on an ILAB 600 Chemistry Analyzer (Instrumentation Laboratory, Bedford, MA, USA). A comparison was then made between the environmental data acquired during scene investigation and the autopsy findings.

### 2.2. Literature Review

A rapid review of the literature was performed through PubMed NCBI and Scopus search engines. The keywords “Wischnewsky Spots” or “Wischnewsky Lesions” were used as mesh terms. The searching included English papers published in the period 2001–2021. All types of papers were included, i.e., case reports, reviews, retrospective analyses, experimental studies. Papers concerning this subject, which were found through citations, were added. Exclusion criteria were narrative papers that only cited WS among the signs of hypothermia without describing practical cases or investigating its pathophysiology. Other exclusion criteria were non-English papers or works published before 2001.

## 3. Results

### 3.1. Cases Presentation

#### 3.1.1. Case 1

An 87-year-old man, who had been missing for a few days, was found dead in an open area on a steep hill. The man had dementia. He was found in an open area, prone on the ground below a hill made up of thick vegetation and trees. The hill presented rough terrain and very cold ambient temperatures. The body presented head trauma with multiple injuries, such as bruises spread over the whole body.

#### 3.1.2. Case 2

An 81-year-old woman was found dead in an area near a dry river with clayey soil, showing widespread abrasions on the lower limbs. The woman had dementia under drug treatment and was in a nursing home from which she had escaped. The body was found below a railway bridge. The scene showed clayey soil with a vast isolated countryside, surrounded by trees and various shrubs. 

#### 3.1.3. Case 3

An 85-year-old man was found dead in a river with various head injuries (post-mortem skin lacerations due to prolonged immersion in water) and bruises on his lower limbs. The man had psychosis with schizophrenia and delusions, for which he was in a nursing home, from which he had left several times in the previous months. The video cameras showed the man’s escape in the night toward the path that led to a river about 80 m from the nursing home. The body was found the next morning naked in the riverbed.

### 3.2. Literature Review

The literature review led to the selection of 23 papers, published over the period 2001–2021 [4,5,6,7,8,9,10,11,12,13,14,15,16,17,18,19,20,21,22,23,24,25]. The review included 12 case reports, 4 retrospective analyses, 3 experimental models, 2 immunohistochemical analyses, 1 review, 1 commentary. 

### 3.3. Scene Investigation and Autopsy Findings

In the cases described, the scene analysis showed open environments with cold temperatures (Table 1). 

#### 3.3.1. Case 1

In Case 1, it was a steep hill located at great heights. The scene showed an impervious path with stones and rocks about 9 m perpendicularly from the point of discovery of the body. The terrain in this area was steep with roughness. Therefore, it was reasonable to assume that the man in this area and at this height could have accidentally lost his balance. At autopsy, the body showed widespread bruising with no evidence of fractures and a head injury with slight subarachnoid hemorrhage. The heart presented some whitish areas on the left ventricle compatible with a past ischemia (chronic ischemic heart disease). No signs of an acute cardiac event were found. The examination of the stomach revealed hyperemic areas, with red-brownish hemorrhages and petechiae with a diameter ranging between 0.1 and 0.4 cm located on the surface of the gastric folds. The reconstruction of the forensic data revealed that the man had reached this inaccessible area and accidentally lost his balance by falling into free fall and hitting the rocks placed on the ground. This impact probably caused the subject’s loss of consciousness, which remained with reduced mobility for a long time in an open environment with low temperatures and consequent hypothermia.

#### 3.3.2. Case 2

In Case 2, the scene was a steep area located near a river where the body was found. The route was characterized by a road that started from the nursing home and, crossing the tracks, ended up to the banks of the stream through a cliff. The woman’s head rested on ground that showed about 7 cm of water. The external temperature in the previous days was about 0–2 °C. Autopsy showed multiple excoriations with bruising of the knees (frost erythema) and fracture on the XII thoracic vertebra compatible with a fall from height. The analysis of the heart showed no significant changes. The examination of the stomach revealed an accentuated vascularization of the organ externally and on the gastric mucosa with microhemorrhages characterized by red-brownish spots, 0.1–0.2 mm in size with surrounding hyperemia of the folds, accentuated on the bottom gastric and other areas with a purplish appearance of about 2 cm, similar to ulcer. Autopsy data, in accordance with the data acquired on the scene, suggested that the woman had remained immobilized in the cold, trying to drag herself with the help of the upper limbs due to the vertebral fracture.

#### 3.3.3. Case 3

**Figure 1:**
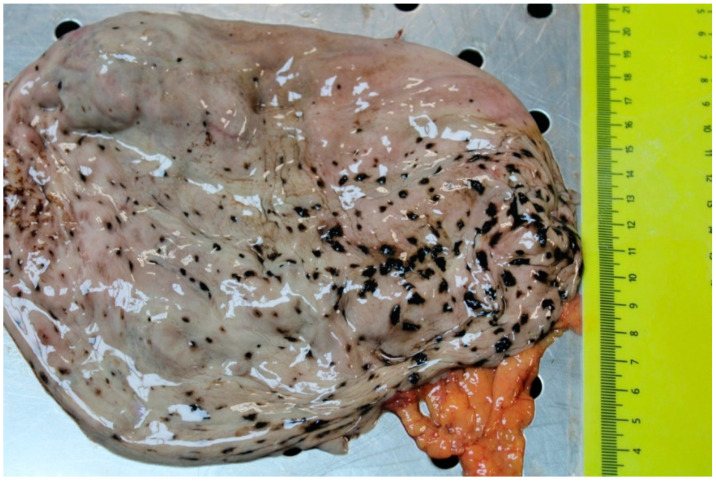
Macro hemorrhages of about 0.4–0.5 cm in diameter spread over the entire surface of the gastric mucosa (Case 3).

**Figure 2:**
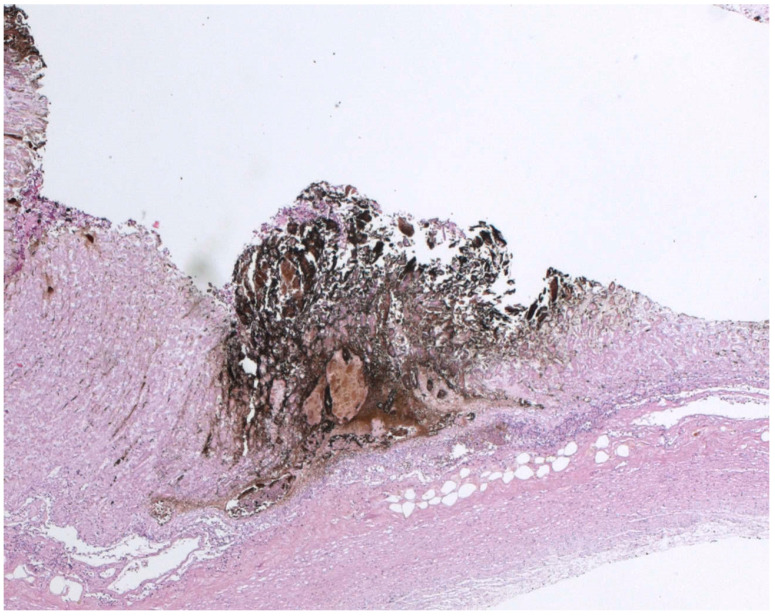
Microscopic analysis of WS of significant erosions (H+E staining).

In Case 3, the body was placed in countryside. The body was found inside a river with temperatures of about 4° at the time of the inspection. Meteorological data from the previous evening confirmed a temperature below 0°. Autopsy showed signs of hypothermia, such as frost erythema in the lower limbs, and internal body signs, such as hemorrhage of the synovial membranes. The autopsy also showed mild subarachnoid hemorrhage in the frontal region, heart contracted in systole with moderate coronary atheromasia, fracture of the fourth thoracic vertebra and spots on the stomach consisting of black macro hemorrhages of about 0.4–0.5 cm in diameter spread over the entire surface of the gastric mucosa (Figure 1 and Figure 2).

#### 3.3.4. Analysis of Cases

**Figure 3:**
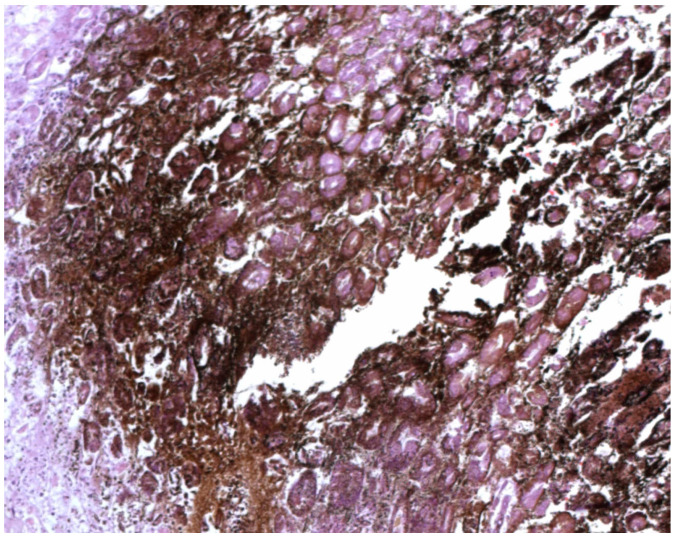
Microscopic analysis of WS with areas of glandular accumulation and lymphoplasmacellular infiltrate (H+E staining).

Autopsy showed, in all cases, a reduced mobility caused by head trauma in Case 1 and by new vertebral fracture in Cases 2 and 3. The autopsy suggested that these traumas were not of sufficient severity to cause death. Gross and histological findings on the stomach confirmed the presence of WS on the folds of the gastric mucosa. From our experience, documented through the three cases reported, it is evident that the spots can present themselves with various sizes (micro or macrohemorrhagic appearance), color shades (from red purple to black) and localization on various areas of the mucosa. Microscopic investigations in our cases confirmed the presence of areas of glandular accumulation with a hemorrhagic appearance and lymphoplasmacellular infiltrate (Figure 2 and Figure 3). No other relevant cause of death was evident at autopsy. These data, in agreement with other signs found at autopsy, with histopathological data and, above all, with the circumstantial data on the scene, confirmed that the death was due to hypothermia with reduced mobility for a long time due to the trauma. Toxicological investigations were negative in all cases.

## 4. Discussion

The literature review showed that WS have a variable incidence in hypothermia cases (43–100%). Wischnewski reported a percentage of 91% [3]; Mant observed in 86% of cases [26]; Clark et al. in 73% [16]; Mizukami et al. in 43.5% [27]; Haba et al. in 87% [28]; Bright et al. in 92–100% [18]; Dickinson in 56.6% [6].

WS spots have been described as multiple hemorrhages, of a dark blackish color, present on the surface of the gastric mucosa, in particular on the apex of the gastric folds in variable numbers (up to 100), with oval or punctiform shape, ranging in diameter from 1 mm to 2 cm. Microscopic investigations found in our review suggested that WS cannot be considered as simple ulcerations of the mucosa but as peculiar, multiform and multidimensional injuries of the gastric folds, characterized by lymphoplasmacellular infiltrate with surrounding erosions [17]. Besides, Tsokos et al. have not found a correspondence of WS with ulcerations, describing them in HE (hematoxilin-eosin) sections as amorphous material with a pink color with the possibility of lymphoplasmacellular infiltrate and infarcts of the surrounding mucous glands [23]. The immunohistochemical analysis showed a positivity to antihemoglobin, so some authors proposed a hypothesis based on the initial hemorrhage of the gastric mucous glands with subsequent autolysis, destruction of red blood cells and hematinization of hemoglobin giving the typical blackish color. Almeida et al. confirmed the presence of hematin deposits in the WS observed in monkeys [5]. Clark et al. have described glandular and intestinal accumulation of blackish material with hemorrhagic aspects, excluding that WS are simply mucosal ulcers [16].

**Figure 4:**
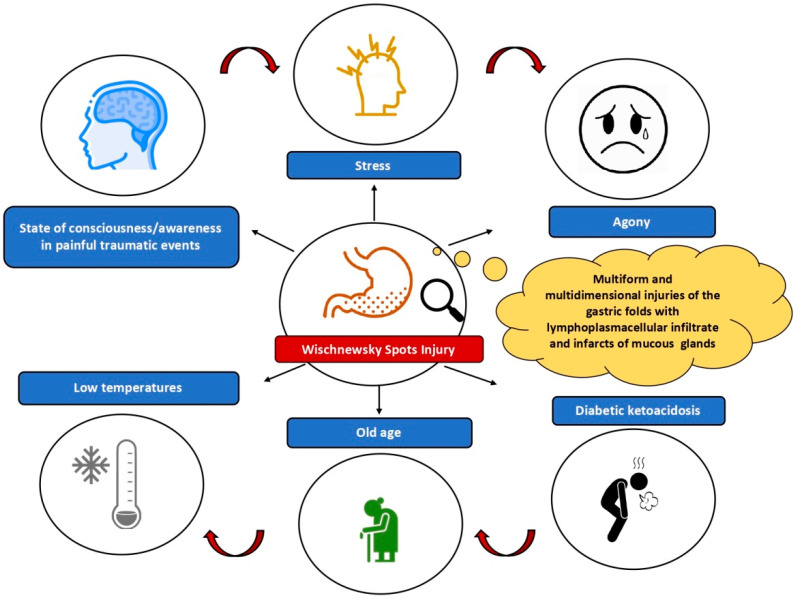
Multifactorial pathophysiology of WS.

The review demonstrated a complex and multifactorial pathophysiology of WS (Figure 4).

In 2020, Yang et al. hypothesized a role of hypothermia in the stimulation of acid secretion associated with WS [8]. The authors compared rat gastric tissues kept in culture at low temperatures with a control group. The experiment showed an increase in the mRNA expression of gastrin and pepsin C, resulting in mucosal and vascular gastric injury, increase in gastric Hydrogen (H^+^), Potassium (K^+^) -ATPase pump protein and resulting in morphological changes in parietal cells. The experimental model proved with certainty that lowering the temperature stimulates the expression of gastrin and pepsin C, affecting the integrity of gastric mucosa and causing vascular damage on the gastric wall [8]. Therefore, a role of gastrin and low pH in the pathogenesis of this phenomenon is clear.

The literature has reported WS in various cases, not only of hypothermia, but also of diabetic ketoacidosis (DKA). Clark et al. reported a retrospective study in deaths from DKA with incidence of WS in approximately 40% of cases [16], suggesting that WS are a non-specific finding that can be present in both causes of death. McCarthy et al. described a possible association between hypothermia, alcohol abuse and DKA with formation of WS in these cases due to metabolic derangement [9]. According to Tse et al., during hypothermia, there is an increase in catecholamines with inhibition of insulin release, increase in glycogenolysis and gluconeogenesis with ketoacidosis and hyperglycemia [11]. However, urinary catecholamines were not assayed in the three cases because the test was not available to the authors. For this reason, the finding of WS in deaths due to hypothermia and DKA could be due to a common mechanism with reciprocal effects. In these cases, an increase in post-mortem vitreous glucose and β-hydroxybuyrate can also be found [11]. It is clear that WS can occur in DKA without co-existing hypothermia, so it could be that hypothermia can precipitate DKA. However, based on our knowledge at this moment, there is no certain evidence of this mechanism. Among the other causes of death, Ortmann et al. reported a gunshot suicide with WS on gastric mucosa [14]; Duval et al. a case of hypothermia with impalement and reduced mobility; Kanchan et al. also found the spots in fatal burn injuries [8]. Zivković et al. showed that WS can be visible even in bodies with post-mortem changes, reporting them in two partially mummified bodies [19]. Many of the cases reported in the literature review proved that various factors, such as decrease in body temperature, but also other extrinsic and intrinsic factors, including physical and psychological stress, agony, metabolic comorbidities and *causa mortis*, may be associated with WS. Several in vivo experimental models were carried out to evaluate the role of physical stress with WS finding on gastric mucosa. In 1984, Vincent et al. reported that the reduction in stress in animals kept at cold temperatures resulted in a reduction in the number and size of WS, hypothesizing a crucial role for stress-related gastric acid secretion [29]. In particular, the model of Bright et al. (2013), with 42 anesthetized rats, and, therefore, unconscious, is emblematic from this point of view, as it did not find any lesions despite death being due to hypothermia because the animals were placed in a cold environment [20]. Bright et al. underlined the role of physical and psychological stress at the moment of death, related to physical impairment, pain and sense of imminent death [18,20]. Similar considerations apply to the Landeira-Fernandez model, which confirmed the role of physical and psychological stress and the active state of consciousness at the time of death with the incidence of WS [25]. In 2004, the author developed an animal model to test the effects of stress on WS appearance by comparing anesthetized rats and conscious rats exposed to cold [25]. The study showed that conscious animals developed more extensive and severe injuries on the gastric mucosa than unconscious ones, although the external temperature was the same. Therefore, the level of consciousness appears to be decisive in WS appearance. 

Of course, it is necessary to consider that these models were carried out on animals and that, therefore, the application of the same conditions on humans could show additional variables affecting the WS phenomenon. In this regard, the case reports reported in Table 1 all documented the finding of WS in particularly stressful conditions, with a long period of agony, including reduced mobility from traumatic fractures, impalement and even a case of thermal energy injuries [15]. The comparison with our series confirmed the presence of hypothermia in all three cases associated with a long period of agony (demonstrated by the finding of lesions not compatible with sudden death and typical clots related to this phenomenon), with reduced mobility attributable to traumatic fractures. Therefore, based on the reported cases and the review data, we hypothesize that low temperatures, along with injuries causing intense agony or physical/psychological stress, may favor the development of WS. In this sense, it cannot be excluded that the development of these lesions follows pathophysiological mechanisms that are similar with gastric stress ulcers. For this reason, we can hypothesize a determining role of the cortisol axis and of the production of catecholamines as a response to stress in the onset of WS. Finally, we underline that the case reports described in the literature concerned people aged 40 or over, and in the literature, no cases of WS were found in children or young adults, except for only one boy of 14- and a 21-year-old man who died from diabetic ketoacidosis but not for hypothermia. These data would confirm the hypothesis proposed in the animal experimental model of Kiang-Ulrich et al., which proved greater resistance to low temperatures and to WS development in younger rats [30], thus developing fewer spots. Besides, the authors showed that 3-month-old rats were less likely to develop WS than 12- and 24-month-old rats exposed to the same environmental conditions [30] by placing 42 anesthetized rats in close contact with an ice box. Even in the cases reported by our experience, the subjects were of advanced age. This phenomenon could, therefore, be age related, both due to intrinsic resistance to low temperatures associated with age, but also due to the lower probability that cases of hypothermia will occur in young people compared to the elderly, who show higher risk factors. Certainly, other studies will be necessary to confirm the pathophysiological data emerging from this rapid review with statistical significance, especially through the analysis of case reports or autopsy cases of hypothermia, post-mortem studies with histopathology and immunohistochemical investigations of the gastric mucosa, as well as in vitro/in vivo experimental animal models.

## 5. Conclusions

The data emerging from our review suggest that WS are lesions with variable incidence with greater probability in cases of:

- Hypothermia;
- Physical stress with prolonged agony caused by concomitant and particularly painful traumatic events (impalement, traumatic fractures with reduced mobility);
- Activation of gastrin and pepsinogen correlated to low temperatures;
- Old age;
- Diabetic ketoacidosis.
- In this regard, we encourage the publication of further forensic cases of hypothermia or WS finding by suggesting, for the purpose of homogeneity between the studies, to:
- Describe an accurate analysis of the scene where the body was found (rivers, sea, mountains, cold environments, etc.) reporting the ambient temperature;
- Report patient’s age, circumstantial data, hide and die phenomena, any stress factors at the time of death, previous comorbidities;
- Describe macroscopic and microscopic autopsy data, with any other signs of hypothermia;
- Perform post-mortem laboratory investigations, including vitreous glucose and β-hydroxybutyrate, to investigate the correlation with diabetic ketoacidosis.

Finally, for forensic purposes, we propose, to always perform an accurate analysis of the gastric mucosa, in all cases of deaths occurred with agony or physical stress. We suggest a careful evaluation of WS that could be potentially confused with autolytic phenomena of the gastric mucosa with risk of underestimation of the phenomenon. Such reports could also be useful for a better reconstruction of the cause of death for judicial purposes.

## Figures and Tables

**Table 1:** Data about autopsy cases reported in our experience with WS.

Case	Sex	Age	Comorbidities	Scene Finding	Main Autopsy Findings
1	M	81	Dementia	Steep hill	Head trauma
2	F	83	Dementia	Impervious area to the banks of a river	Vertebral fracture
3	M	85	Schizophrenia	Recovery of the body in a river	Vertebral fracture

## Data Availability

Not applicable to this article, as no datasets were generated.
